# PDE-4 Inhibition in Sarcoidosis Patients: A Retrospective Single-Center Analysis of 51 Patients

**DOI:** 10.3390/ph18111729

**Published:** 2025-11-14

**Authors:** Martin Elias Feineis, Charlott Terschluse, Louis Jouanjan, Daniel Soriano, Prerana Agarwal, Jonas Schupp, Joachim Müller-Quernheim, Daiana Stolz, Björn Christian Frye

**Affiliations:** 1 Department of Pneumology, Medical Center–University of Freiburg, Faculty of Medicine, University of Freiburg, 79106 Freiburg, Germany; martin.feineis@gmail.com (M.E.F.); charlott.terschluse@mailbox.org (C.T.); louis.jouanjan@uniklinik-freiburg.de (L.J.); daniel.soriano@uniklinik-freiburg.de (D.S.); schupp.jonas@mh-hannover.de (J.S.); joachim.mueller-quernheim@uniklinik-freiburg.de (J.M.-Q.); daiana.stolz@uniklinik-freiburg.de (D.S.); 2 Department of Diagnostic and Interventional Radiology, Medical Center–University of Freiburg, Faculty of Medicine, University of Freiburg, 79106 Freiburg, Germany; prerana.agarwal@uniklinik-freiburg.de; 3 Clinic for Respiratory Medicine and Infectious Diseases, Hannover Medical School, 30625 Hannover, Germany

**Keywords:** sarcoidosis, roflumilast, lung function, PDE-IV inhibitors

## Abstract

**Background**: Several sarcoidosis patients require treatment with corticosteroids to prevent organ damage and control symptoms. However, corticosteroids are associated with numerous side effects and can be detrimental to patients if used long-term. Roflumilast is approved for the treatment of chronic obstructive pulmonary disease (COPD) and has been studied with positive results in patients with fibrosing sarcoidosis. Due to its mode of action, it targets proinflammatory and profibrotic pathways involved in sarcoidosis and could be a suitable medication for sarcoidosis. **Methods**: We retrospectively analyzed a cohort of 51 sarcoidosis patients treated with Roflumilast off-label between 2010 and 2020 at the Department of Pneumology, University Hospital Freiburg. Medical records, lung function, and laboratory results were reviewed. **Results**: Of the 51 patients, 33 patients received Roflumilast for at least 6 months, whereas 18 discontinued treatment, mostly due to mild to moderate gastrointestinal side effects (*n* = 7). No severe adverse events were observed. Patients on Roflumilast were less likely to have a decrease in FEV1 of more than 10% of their mean FEV1 compared to patients without Roflumilast (OR = 0.2; 95% CI 0.08–0.5). Escalation of therapy was documented in 49/97 (51%) of ambulatory visits for patients taking Roflumilast compared to 100/144 (69%) for patients without Roflumilast (OR = 0.45; 95% CI 0.26–0.76). **Conclusions**: Sarcoidosis patients receiving Roflumilast had less lung function loss and were less likely to require therapy escalation. Roflumilast could be a therapeutic option in sarcoidosis.

## 1. Introduction

Sarcoidosis is a granulomatous disease of unknown origin that can affect almost every organ, with a predilection for the lung and intrathoracic lymph nodes [1]. Corticosteroid therapy is well established and recommended by current guidelines as first-line sarcoidosis treatment if required to prevent organ damage or to control symptoms [2,3]. Lacking clinical remission, corticosteroid side effects or long-term corticosteroid therapy represent common indications for starting a second-line therapy with disease-modifying antirheumatic drugs such as methotrexate and azathioprine to allow reducing steroid dose and ideally cessation [2,3]. TNF-α inhibitors, such as infliximab, are reserved for severe or refractory disease as third-line therapeutic options [2,4]. However, in Germany, there is currently no medication officially approved for the treatment of sarcoidosis, resulting in a widespread, formal off-label use of drugs, especially in steroid-refractory disease.

Recent data from a Dutch multicenter randomized controlled trial demonstrated that methotrexate may serve as an effective first-line alternative to prednisone in pulmonary sarcoidosis, providing comparable efficacy with a more favorable safety profile [5]. These findings support an emerging shift toward earlier integration of steroid-sparing regimens in sarcoidosis management. Several analyses emphasize the need for steroid reduction due to their severe side effects [6,7]. Notably, the Food and Drug Administration (FDA) allowed corticosteroid reduction in chronic sarcoidosis as a pivotal and primary endpoint in a clinical trial (NCT03824392).

However, long-term use of corticosteroids and immunosuppressants is associated with a wide range of adverse effects, highlighting the need for alternative therapeutic options. Newly arising and promising approaches include biologic immunomodulators such as efzofitimod, which targets neuropilin-2 on activated immune cells and has demonstrated clinical benefits in pulmonary sarcoidosis [8,9] or the Janus kinase (JAK) inhibitor tofacitinib with promising efficacy, particularly in cutaneous sarcoidosis [10,11].

Several immunosuppressive agents currently used in the management of sarcoidosis were initially developed for other diseases and have since been repurposed. In this study, we sought to assess the therapeutic potential of roflumilast, a selective phosphodiesterase-4 (PDE4) inhibitor currently approved for the treatment of chronic obstructive pulmonary disease (COPD) [12].

PDE-4 is an enzyme highly expressed in neural tissue, smooth muscle, skin, and various immune cells, including macrophages, T cells, neutrophils, eosinophils, and dendritic cells [13]. Inhibition of PDE-4 results in increased intracellular cyclic adenosine monophosphate (cAMP) levels, which downregulate proinflammatory cytokines such as TNF-α, IFN-γ, and IL-17, upregulate the anti-inflammatory cytokine IL-10, and may exert antifibrotic effects through modulation of fibroblast activation and collagen deposition [13,14,15].

Given that sarcoidosis involves both inflammation and fibrosis, PDE-4 inhibition could be a suitable therapeutic option in sarcoidosis by targeting the key cytokines involved. Recently, two clinical trials have demonstrated the efficacy of nerandomilast (a specific PDE-4B inhibitor) in idiopathic pulmonary fibrosis (IPF) [16] and progressive pulmonary fibrosis (PPF) [17]. In the latter one, patients with PPF on the background of inflammatory diseases were included, of which only 17 (1.4% of the study population) had sarcoidosis.

Integration of novel agents such as roflumilast into existing therapeutic approaches may help to further reduce corticosteroid exposure and reduce the risk of immunosuppressive therapies.

However, clinical data on roflumilast use in sarcoidosis remain limited. In a placebo-controlled clinical trial by Baughman et al., involving 28 patients with pulmonary fibrosis secondary to sarcoidosis, roflumilast reduced the risk of acute disease exacerbations and improved quality of life, as measured by the King’s Sarcoidosis Questionnaire (KSQ) Lung Score [18]. However, the study population was small, and only 10 patients completed 12 months of treatment [18].

The aim of this retrospective analysis is to expand current knowledge on the effect of roflumilast in the broader sarcoidosis population by analyzing the disease course of 51 patients treated with roflumilast.

## 2. Results

### 2.1. Patient Characteristics

**Figure 1:**
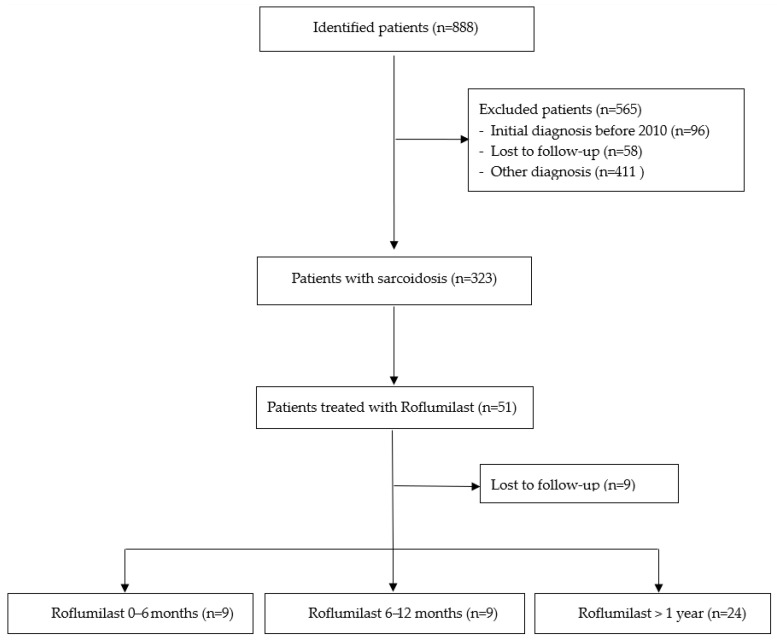
Consort Diagram.

The patient selection process is shown in Figure 1. 51 patients were included in the analysis, of whom 33 (65%) received roflumilast treatment for more than six months. Eighteen patients who discontinued roflumilast before 6 months served as a comparative control group. Discontinuation was mostly due to mild gastrointestinal side effects, and the threshold to withdraw the medication was low given its off-label use.

Baseline characteristics, including disease manifestations, age, baseline therapy, pulmonary function, and serological parameters, are summarized in Table 1. No significant differences were observed between the two groups. Roflumilast was started as monotherapy in 18/51 patients, whereas the others received roflumilast in combination with mainly corticosteroids. The mean treatment duration among patients who continued therapy was 2.1 years (SD 1.3), with a median follow-up of 5 years (IQR, 3–7 years). Lung function parameters, obstructive phenotype, and smoking status were evenly distributed between groups. Notably, both groups demonstrated a relatively high mean body mass index (BMI), likely reflecting the selection of overweight patients, given that weight loss is a known side effect of roflumilast.

### 2.2. Clinical Outcomes During Follow-Up

Patients receiving roflumilast had a significantly lower risk of disease progression (Table 2). Specifically, these patients were less likely to experience a decline in FEV1 of more than 10% compared to their mean FEV1 (mean FEV1 81.5% pred.) (OR 0.20, 95% CI 0.08–0.50, *p* < 0.01) and had a lower likelihood of clinically assessed disease progression (OR 0.38, 95% CI 0.19–0.72, *p* < 0.01). New organ involvement was reported in 3/142 (2%) of visits while taking roflumilast, compared to 9/172 (5%) while not taking roflumilast. However, this effect was not significant (OR 0.39; 95% CI 0.10–1.74, *p* = 0.24).

The need for therapy escalation (Table 3) was significantly lower in the roflumilast group (49/97 [32%] vs. 100/144 [69%]; OR 0.45, 95% CI 0.26–0.76, *p* < 0.01). Subgroup analysis showed a numerical reduction in (i) corticosteroid dose increase (13/97 [9%] vs. 25/144 [17%]; OR 0.73, 95% CI 0.34–1.54, *p* = 0.47) and (ii) initiation of a second- or third-line therapy (31/97 [32%] vs. 53/144 [37%]; OR 0.81, 95% CI 0.47–1.39, *p* = 0.49), as well as a significant reduction in the need for corticosteroid pulse therapy (5/97 [5%] vs. 22/144 [15%]; OR 0.30, 95% CI 0.11–0.83, *p* < 0.05).

Therapy de-escalation (Table 3) was not significantly associated with roflumilast use. De-escalation occurred in 43/97 (44%) of visits while taking roflumilast and 59/144 (41%) of visits without roflumilast. Steroid discontinuation rates were identical (6%) in both groups.

### 2.3. Lung Function Parameters, BMI and Serological Parameters

**Figure 2:**
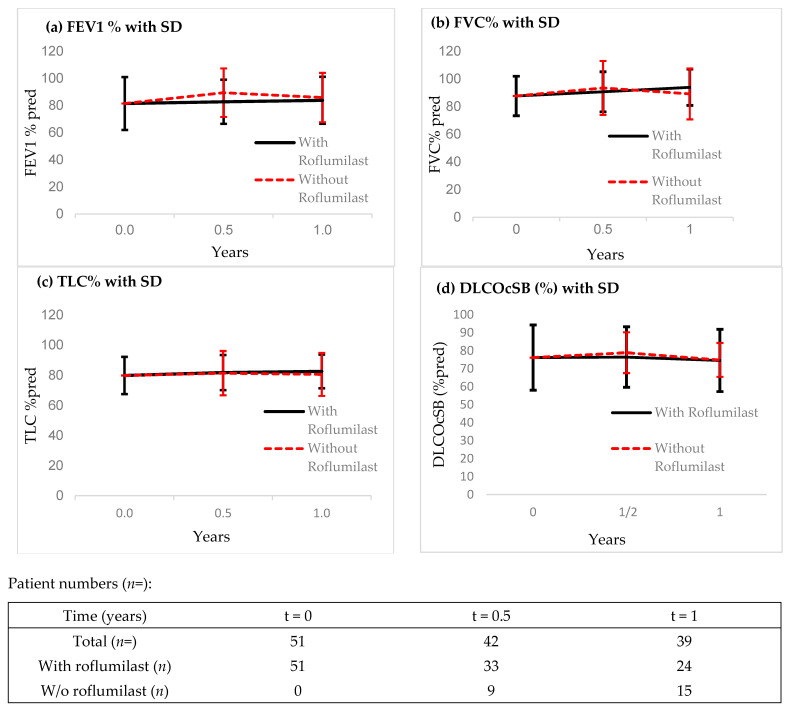
(**a**–**d**): Lung function parameters with or without taking roflumilast (with standard deviation).

No significant changes in lung function parameters (Figure 2a–d) or serological markers of disease activity and BMI (see Supplementary Material Figure S1) were observed following the initiation of roflumilast, and values remained comparable between groups.

### 2.4. Subgroup Analyses

Subgroup analyses comparing disease progression by smoking status, CT-verified fibrotic versus non-fibrotic phenotype, and airway versus no airway involvement (see Supplementary Material Tables S1–S3) indicated a numerical benefit of roflumilast across all three subgroups in terms of disease progression prevention. The numerical effect was most apparent among patients with fibrotic disease, where progressive disease was observed in 1 of 47 (2%) visits during roflumilast treatment, compared with 11 of 47 (23%) visits when patients were not receiving the drug. Similarly, patients with fibrotic disease experienced fewer visits with a ≥10% decline in FEV1 relative to the mean FEV1, while on roflumilast (3 of 47 [6%] vs. 20 of 47 [42%] of visits).

### 2.5. Adverse Effects

Most commonly, patients discontinued Roflumilast due to gastrointestinal disorders (14%). Other reported adverse effects included anxiety and sleep disturbances (Table 4). All adverse events were mild to moderate in severity. Given the off-label administration of roflumilast, threshold for discontinuing roflumilast was low. No hospitalizations were required, and no severe adverse effects were observed among patients exposed to Roflumilast.

## 3. Discussion

This retrospective analysis adds further evidence for the use of PDE-4 inhibitors in sarcoidosis. To our knowledge, this represents the largest real-world cohort of sarcoidosis patients treated with roflumilast, with a mean treatment duration of more than two years.

The rationale for the off-label use of Roflumilast in sarcoidosis patients was based on the anti-inflammatory and antifibrotic effects of PDE-4 inhibition, which downregulates key cytokines of sarcoidosis [13,15]. These cytokines play an important role in the pathogenesis of sarcoidosis [14] and could, therefore, be suitable as pharmacological targets. The complex mechanism of sarcoidosis, causing inflammation and fibrosis both in the airways and lung parenchyma, provides an additional reason for using drugs targeting multiple cytokines simultaneously [18,19,20]. Previous studies have explored the effects of various PDE-4 inhibitors on cutaneous sarcoidosis [21], their steroid-sparing potential [22], and their role in stabilizing fibrotic sarcoidosis [18]. Recent data on nerandomilast, a selective PDE-4B inhibitor, further underline the therapeutic relevance of PDE-4 inhibition [17]. In patients with progressive pulmonary fibrosis, nerandomilast reduced disease progression and prevented lung function decline compared to placebo treatment over a period of 52 weeks [17].

Mechanistically, PDE-4 inhibition increases intracellular cAMP, which can suppress Th17 signaling and modulate macrophage polarization toward an anti-inflammatory phenotype [13,15,23]. Additionally, PDE-4 inhibition may intersect with key fibrotic and inflammatory pathways, including JAK-STAT and TGF-β signaling cascades [13,15], potentially contributing to both anti-inflammatory and anti-fibrotic effects in sarcoidosis.

Our results suggest that roflumilast significantly reduces the likelihood of disease progression, particularly in events of lung function decline and clinically assessed disease worsening. These findings are consistent with the findings by Baughman et al. [18], indicating that PDE-4 inhibitors could play a role in modulating the fibrotic and inflammatory processes involved in sarcoidosis. Moreover, our data supports a potential steroid-sparing effect, as roflumilast therapy was associated with lower rates of corticosteroid escalation and decreased need for additional immunosuppressive therapy.

In 33 patients, roflumilast therapy was added to background immunosuppressive therapy. Discontinuation rates of roflumilast differed between patients with and without background therapy, emphasizing its safety even in combination regimens.

The number of patients receiving immunosuppressive therapies beyond corticosteroids was too low to draw conclusions about the value of roflumilast in these patients; however, based on its pharmacological profile, it could represent an additional option even in those cases.

In contrast to the clinical study by Baughman et al. [18], which focused on the fibrotic phenotype, our patient cohort was not specifically selected and encompasses different clinical, lung functional and radiological phenotypes. Additionally, patients continuing therapy were exposed to roflumilast for longer periods without increased side effects, pointing towards long-term safety. Also, for nerandomilast, background immunosuppressive therapy was allowed in the FIBRONEER trial [16], although explicit data on the safety within this subgroup are not yet published.

As patients with obstructive and mixed ventilatory defects were also included, we used FEV1 as a major lung function parameter to assess lung functional decline. As expected, roflumilast did not significantly affect overall lung function parameters. This aligns with other studies that have struggled to demonstrate lung function improvements in sarcoidosis, suggesting that changes in body plethysmography may not be an adequate endpoint for this disease [24]. The study cohort was also underpowered to detect subtle changes in lung function.

Subgroup analyses based on smoking status, airway involvement, and CT-verified fibrotic versus non-fibrotic phenotype were limited by the cohort size and do not allow detection of statistically meaningful effects or definitive conclusions; therefore, these findings should be considered hypothesis-generating and require confirmation in larger, controlled studies. Nevertheless, it is noteworthy that sarcoidosis patients with radiologically detected airway involvement or fibrotic phenotype seem to benefit more from therapy, whereas active smoking has no influence.

This single-center retrospective analysis has several limitations inherent to its design. Patients were not randomized to treatment, and no uniform inclusion criteria were applied. However, the team deciding on therapies was constant, and as an off-label therapy, only patients without signs of severe organ involvement requiring intense immunosuppression were included.

In addition, access to roflumilast for patients with sarcoidosis remains limited due to its off-label status, which may pose reimbursement challenges and affect treatment availability as well as cost considerations in clinical practice. Nevertheless, the data presented in this study may help support discussions with health insurance providers regarding reimbursement for selected patients.

Furthermore, clinically assessed disease worsening, based on patient-reported symptoms or physician-noted signs from medical records, was not guided by formal criteria, making it subjective and potentially prone to inter-observer variability.

Moreover, as all patients were treated in a single tertiary German center, the cohort’s phenotype distribution (fibrotic vs. non-fibrotic), BMI, and predominantly European ethnicity may not fully represent global sarcoidosis populations. These factors should be considered when extrapolating the results to more diverse patient groups. In addition, the off-label nature of roflumilast use likely introduced a selection bias, as patients with severe or rapidly progressive disease manifestations were generally not treated with this agent. Consequently, the present findings may primarily reflect outcomes in individuals with milder or more stable disease courses, which should be taken into account when assessing the generalizability of the data.

This limits the generalizability of the findings, as the effects of roflumilast may differ in more severe cases of the disease. To reduce the selection bias, we compared patients with maintained therapy to patients who stopped therapy due to side effects, since they are supposed to have a similar disease profile, as evidenced by baseline characteristics. However, lack of efficacy may have contributed to the decision of treatment discontinuation, possibly resulting in overrepresentation of treatment success in patients continuing therapy. The retrospective design did not allow the analysis of patient-reported outcome measures or satisfaction with medication. Disease progression, decline of lung function, or need for increased corticosteroid therapy may reflect patients’ quality of life. Patients and treating physicians were not blinded to therapy, which could influence treatment decisions. On the other hand, given the off-label use, the threshold to stop medication because of perceived side effects may be lower, explaining the relatively high number of patients discontinuing therapy. Our relatively small cohort size precluded the use of mixed-effects or generalized estimating-equation models to account for intra-patient clustering, representing another study limitation. Despite this, roflumilast demonstrated a favorable safety profile, with no severe treatment-related adverse events observed.

## 4. Materials and Methods

### 4.1. Patient Recruitment

We systematically screened patients who had been under medical examination for diagnosed sarcoidosis between 2010 and 2020 at the Department of Pneumology, Medical Center—University of Freiburg, Germany. Out of 888 patients screened for sarcoidosis during the study period, 565 patients did not meet the inclusion criteria and were excluded. We identified 51 patients who had been treated with off-label Roflumilast. Treatment decision was made by the treating physician in accordance with the patient’s wishes and after extensive information on the off-label use and the potential side effects. Roflumilast was generally initiated to reduce or avoid corticosteroid exposure in patients with symptomatic but non-life-threatening sarcoidosis. Clinical data, lung function metrics, and treatment details were retrospectively extracted from medical records and analyzed after pseudonymization. The study was approved by the Ethics Committee of the Medical Faculty of the Albert-Ludwig-University Freiburg (approval number: 24-1284-S1-retro). The requirement for informed consent was waived due to the study’s retrospective nature. The requirements of the Helsinki Declaration on human research were met.

### 4.2. Inclusion and Exclusion Criteria

Patients were included if they had histologically confirmed sarcoidosis and at least one outpatient visit with available lung function parameters, medication data, and laboratory results. Cases with incomplete medical records or insufficient follow-up data were excluded. For the evaluation of potential therapeutic efficacy, the treatment group comprised patients who had received roflumilast for ≥6 months, whereas patients who discontinued therapy prior to 6 months were assigned to the control group.

### 4.3. Treatment Duration, Dosing, and Concomitant Therapies

Ongoing roflumilast treatment was documented at each patient visit, typically every 3–6 months, and used to calculate individual treatment duration. Roflumilast was administered at a standard oral dose of 500 µg once daily after up-titration with 250 µg for 4 weeks, with dose reduction or temporary discontinuation in cases of intolerance or adverse effects. Concomitant therapies, including systemic corticosteroids or other immunosuppressive agents, were permitted and systematically recorded. Treatment cessation and adverse events were also documented and categorized according to severity and duration.

### 4.4. Therapy Escalation and De-Escalation

Therapy escalation was defined as an increase in corticosteroid dose, administration of corticosteroid pulse therapy, or the addition of a second- or third-line therapy. Therapy de-escalation was defined as dose reduction or discontinuation of corticosteroids or immunosuppressants following clinical improvement.

### 4.5. Disease Progression

Disease progression was defined as a decrease in forced expiratory volume in 1 s (FEV1) by >10%, occurrence of new organ manifestations or documentation of clinically relevant worsening as judged by the treating physician. Clinical worsening was assessed retrospectively from patient records based on patient-reported symptoms or signs noted by the treating physician. No formal criteria were applied.

### 4.6. Subgroup Analysis

Disease progression was evaluated across subgroups defined by smoking status (active vs. pooled ex-/never-smokers), CT-verified fibrotic or non-fibrotic phenotype, and presence or absence of airway involvement. CT scans were reviewed to determine fibrotic and non-fibrotic phenotypes as well as airway involvement. Scans of sufficient image quality were analyzed using the Picture Archiving and Communication System (PACS) by a radiologist experienced in thoracic imaging, as previously described (Agarwal, Feineis et al., *ERJ Open Research*, in press [25]). Due to the relatively small cohort size, we were unable to perform mixed-effects or generalized estimating-equation models to account for intra-patient clustering.

### 4.7. Statistical Analysis

Data were collected in a pseudonymized database with access limited to physicians involved in data collection and handling. All analyses were performed with Stat View 5.0. Continuous variables are presented as mean ± standard deviation (SD) or median (interquartile range), as appropriate. Categorical variables are expressed as counts and percentages. Comparisons between groups were made using *t*-tests or Mann–Whitney U tests for continuous variables. Categorical variables were analyzed using Fisher’s exact test. A *p*-value < 0.05 was considered statistically significant. Odds ratios (OR) and 95% confidence intervals (CI) were estimated using binary logistic regression analysis, and statistical significance for ORs was defined as a 95% CI not including 1.0. We calculated only crude odds ratios (ORs) due to the limited cohort size.

## 5. Conclusions

In conclusion, roflumilast may represent a potential treatment option for selected patients with sarcoidosis. Roflumilast-treated patients exhibit a trend towards reduced corticosteroid exposure and less likely disease progression. Even though a single-center retrospective analysis, these results are promising and prospective clinical trials are needed to confirm the hypothesis that roflumilast may represent a beneficial therapeutic option for sarcoidosis patients.

## Figures and Tables

**Table 1:** Clinical features of all patients under Roflumilast treatment (*n* = 51).

	Roflumilast Treatment ≥ 6 Months (*n* = 33)	Roflumilast Treatment < 6 Months (*n* = 18)	Mann–Whitney U-Test/*Fisher’s test* *p*-Value
Baseline characteristics			
Age at diagnosis (mean/range)	45.5 (26–62)	51.3 (27–70)	0.1
Sex (female/male) %	16/17 48	7/11 39	*0.6*
BMI (kg/m^2^) (mean/range)	31.3 (19–47.7)	29.4 (21.3–47.7)	0.3
Smoking (active/ex-smokers)	4/8	1/12	*0.6*
PY (mean)	12.3	14.5	
Other organ involvement:			
Neurologic	1	1	*1*
Eyes	3	2	*1*
Heart	1	1	*1*
Liver	2	1	*1*
Spleen	4	2	*1*
Bone	3	0	*0.5*
Skin	8	3	*0.7*
Initial immunosuppression			
Corticosteroids (N/%) mg/day (SD)	19 (58) 15.8 (11.6)	9 (50) 10.5 (8.1)	*0.8* 0.6
Azathioprine (N/%)	4 (12)	2 (11)	*1*
Methotrexate (N/%)	1 (3)	0 (0)	*1*
Mycophenolate (N/%)	1 (3)	0 (0)	*1*
Abatacept (N/%)	0 (0)	1 (5)	*0.4*
Body plethysmography			
Tiffeneau Index < 0.7 (-n/%)	8 (24%)	4 (22%)	*1*
FEV1% (mean/SD)	81.5 (21.1)	75.6 (17.8)	0.4
FVC% (mean/SD)	88.1 (20.1)	82.5 (19.5)	0.3
TLC% (mean/SD)	81.1 (40.3)	76.1 (36.8)	0.4
DLCO% (mean/SD)	75.6 (18.5)	71.3 (16.7–94)	0.2
Biochemical markers			
sIL2R (mean/SD)	784.5 (549.5)	538.6 (230.8)	0.3
Neopterine (mean/SD)	14.3 (7.7)	15.3 (5.8)	0.9

**Table 2:** Disease progression at ambulatory visits.

Ambulatory Visits	With Roflumilast	w/o Roflumilast	Odds Ratio (95% CI)	*p*-Value (Fisher’s Exact)
N	142	172		
**Disease progression**				
FEV1 decrease > 10% of mean value (N/%)	6 (4%)	31 (18%)	0.20 (0.08–0.50)	*p* < 0.01
Progressive disease (N/%)	15 (11%)	41 (24%)	0.38 (0.19–0.72)	*p* < 0.01
New organ involvement (N/%)	3 (2%)	9 (5%)	0.39; (0.10–1.74)	*p* = 0.24

**Table 3:** Therapy escalation and de-escalation.

Ambulatory Visits with Corticosteroid Therapy	With Roflumilast	w/o Roflumilast	Odds Ratio (95% CI)	*p*-Value (Fisher’s Exact)
**N**	**97**	**144**		
**Therapy escalation (N/%)**	49 (51%)	100 (69%)	0.45; (0.26–0.76)	*p* < 0.01
Increase in OCS dose (N/%)	13 (13%)	25 (17%)	0.73; (0.34–1.54)	*p* = 0.47
Corticosteroid Pulse therapy (N/%)	5 (5%)	22 (15%)	0.30; (0.11–0.83)	*p* < 0.05
Additional 2nd/3rd line treatment (N/%)	31 (32%)	53 (37%)	0.81; (0.47–1.39)	*p* = 0.49
**Therapy de-escalation (N/%)**	43 (44%)	59 (41%)	0.79; (0.68–1.93)	*p* = 0.69
Reduction in steroid dose (N/%)	35 (36%)	49 (34%)	1.15; (0.62–2.11)	*p* = 0.78
Steroid discontinued (N/%)	6 (6%)	8 (6%)	1.14; (0.37–3.44)	*p* = 1.00
Additional 2nd/3rd line treatment discontinued (N/%)	2 (2%)	2 (1%)	1.52; (0.21–10.99)	*p* = 1.00

**Table 4:** Reasons for stopping Roflumilast treatment.

	Patient Number
**Side effects:**	
Gastrointestinal disorders	7/51
Circulatory disorders	2/51
Anxiety	1/51
Sleeping disorders	2/51
**Others:**	
Alternative drug	1/51
All medications discontinued	4/51
Not specified	10/51

## Data Availability

The data presented in this study are available on request from the corresponding author, provided that no statutory or regulatory provisions contradict this. Deposition of data in a central repository is not covered by the ethics approval.

## Supplementary Materials

The following supporting information can be downloaded at: https://www.mdpi.com/article/10.3390/ph18111729/s1.

## Abbreviations

The following abbreviations are used in this manuscript:

BMI	Body mass index
COPD	Chronic obstructive pulmonary disease
DLCO	Diffusing Capacity of the Lungs for Carbon Monoxide
FEV1	Forced expiratory volume in one second
FVC	Forced vital capacity
IPF	Idiopathic pulmonary fibrosis
OCS	Oral corticosteroid
PDE-4	Phosphodiesterase 4
PPF	Progressive pulmonary fibrosis
sIL2R	Soluble Interleukin-2 Receptor
suppl	Supplementary material
