# Supplementary material for: PDE-4 Inhibition in Sarcoidosis Patients: A Retrospective Single-Center Analysis of 51 Patients
Source: Pharmaceuticals (Basel). 2025 Nov 14;18(11):1729. doi: 10.3390/ph18111729 (PMC12654997; doi:10.3390/ph18111729)
Supplement: Supplementary file 1 [file pharmaceuticals-18-01729-s001.zip › pharmaceuticals-3935715-supplementary.pdf]

Article

PDE-4 Inhibition in Sarcoidosis Patients: A Retrospective Single-Center Analysis of 51 Patients

Supplementary material:

Figures:

Figure S1 a-b) BMI and sIL2-R with or without taking roflumilast (with standard deviation).

Tables:

Table S1: Disease Progression by Active Smoking Status

Table S2: Disease progression in patients with CT verified fibrotic vs. non-fibrotic disease

Table S3: Disease Progression in Patients with Airway vs. Non-Airway Involvement

Figures:

Figure S1 a-b) BMI and sIL2-R with or without taking roflumilast.

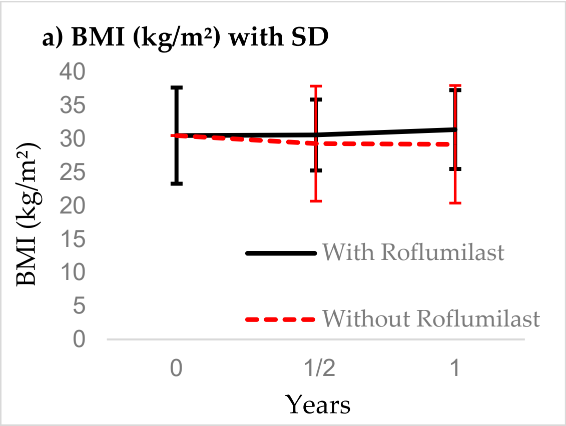

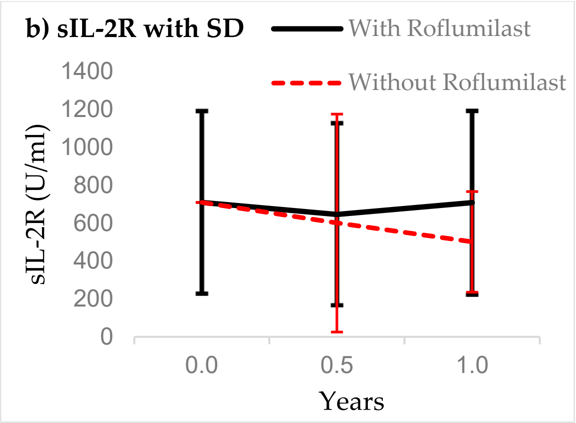

Patient numbers (n=):

Time (years)	t = 0	t = 0.5	t = 1
Total (n=)	51	42	39
With roflumilast (n)	51	33	24
W/o roflumilast (n)	0	9	15

Tables:

Table S1: Disease Progression by Active Smoking Status

	Active smokers (n=4)		Non active smokers (n=29)	
**Ambulatory visits, total** N	38		276	
	**With roflumilast**	**w/o Roflumilast**	**With roflumilast**	**w/o Roflumilast**
**Ambulatory visits (N)**	22	16	119	157
**Disease progression**				
- FEV1 decrease > 10% of mean value (N/% (95% CI)	4 (11%) (5.1–40.2)	8 (21%) (0–20.6)	31 (11%) (18.2–34.9)	79 (29%) (42.4–58.2)
- Progressive disease (N/%) (95% CI)	1 (3%) (0.1–22.8)	2 (5%) (0–20.6)	14 (5%) (6.7–18.9)	39 (14%) (18.3–32.3)
- New organ involvement (N/%)	1 (3%) (0.1–22.8)	1 (3%) (0–20.6)	2 (1%) (0.2–5.8)	8 (3%) (2.2–9.8)

Table S2: Disease progression in patients with CT verified fibrotic vs. non-fibrotic disease

	Fibrotic (n=4)		Non fibrotic (n=29)	
**Ambulatory visits, total** N	47		267	
	**With roflumilast**	**w/o Roflumilast**	**With roflumilast**	**w/o Roflumilast**
**Ambulatory visits (N)**	15	32	126	141
**Disease progression**				
- FEV1 decrease > 10% of mean value (N/%) (95% CI)	3 (6%) (4.3–48.1)	20 (42%) (43.7–79.1)	32 (12%) (18.1–33.7)	67 (25%) (38.7–56.4)
- Progressive disease (N/%) (95% CI)	1 (2%) (0.2–31.9)	11 (23%) (18.6–53.2)	14 (5%) (6.2–17.5)	30 (11%) (15.0–28.9)
- New organ involvement (N/%) (95% CI)	0 (0%) (0–21.8)	1 (2%) (0.1–16.2)	3 (1%) (0.5–6.9)	8 (3%) (2.5–10.9)

Table S3: Comparison of disease progression in patients with CT-verified airway involvement versus no airway involvement.

	Airway involvement (n=8)		No Airway involvement (n=25)	
**Ambulatory visits, total** N	81		233	
	**With roflumilast**	**w/o Roflumilast**	**With roflumilast**	**w/o Roflumilast**
**Ambulatory visits (N)**	35	46	106	127
**Disease progression**				
- FEV1 decrease > 10% of mean value (N/%)	6 (7%) (6.6–33.6)	23 (28%) (34.8–65.2)	29 (12%) (19.0–37.0)	64 (27%) (41.5–59.2)
- Progressive disease (N/%)	3 (4%) (1.8–23.1)	8 (10%) (7.7–32.7)	12 (5%) (5.9–18.9)	33 (14%) (18.8–34.2)
- New organ involvement (N/%)	0 (0%) (0–10.0)	2 (2%) (0.5–14.8)	3 (1%) (0.6–7.9)	7 (3%) (2.2–11.0)
